# Metabolomics Profiling of Age-Associated Metabolites in Malay Population

**DOI:** 10.1155/2023/4416410

**Published:** 2023-02-04

**Authors:** Jen Kit Tan, Siti Nor Asyikin Zakaria, Geetha Gunasekaran, Nur Fathiah Abdul Sani, Muhammad Luqman Nasaruddin, Faizul Jaafar, Zulzikry Hafiz Abu Bakar, Ahmad Imran Zaydi Amir Hamzah, Khairun Nain Nor Aripin, Mohd Dzulkhairi Mohd Rani, Nor Azila Noh, Hanafi Ahmad Damanhuri, Musalmah Mazlan, Suzana Makpol, Wan Zurinah Wan Ngah

**Affiliations:** ^1^Department of Biochemistry, Faculty of Medicine, Universiti Kebangsaan Malaysia, Kuala Lumpur, Malaysia; ^2^Jeffrey Cheah School of Medicine & Health Sciences, Monash University Malaysia, Selangor, Malaysia; ^3^Faculty of Medicine and Health Sciences, Universiti Sains Islam Malaysia, Kuala Lumpur, Malaysia; ^4^Department of Biochemistry and Molecular Medicine, Faculty of Medicine, Universiti Teknologi MARA, Selangor, Malaysia

## Abstract

Aging is a complex process characterized by progressive loss of functional abilities due to the accumulation of molecular damages. Metabolomics could offer novel insights into the predictors and mechanisms of aging. This cross-sectional study is aimed at identifying age-associated plasma metabolome in a Malay population. A total of 146 (90 females) healthy participants aged 28–69 were selected for the study. Untargeted metabolomics profiling was performed using liquid chromatography-tandem mass spectrometry. Association analysis was based on the general linear model. Gender-associated metabolites were adjusted for age, while age-associated metabolites were adjusted for gender or analyzed in a gender-stratified manner. Gender-associated metabolites such as 4-hydroxyphenyllactic acid, carnitine, cortisol, and testosterone sulfate showed higher levels in males than females. Deoxycholic acid and hippuric acid were among the metabolites with a positive association with age after being adjusted for gender, while 9(E),11(E)-conjugated linoleic acid, cortisol, and nicotinamide were negatively associated with age. In gender-stratified analysis, glutamine was one of the common metabolites that showed a direct association with age in both genders, while metabolites such as 11-deoxy prostaglandin F2*β*, guanosine monophosphate, and testosterone sulfate were inversely associated with age in males and females. This study reveals several age-associated metabolites in Malays that could reflect the changes in metabolisms during aging and may be used to discern the risk of geriatric syndromes and disorders later. Further studies are required to determine the interplay between these metabolites and environmental factors on the functional outcomes during aging.

## 1. Introduction

Aging is characterized by a decline in physical fitness and physiological functions over a lifetime. The accumulation of diverse deleterious events, including oxidative stress, mutations, and telomere shortening, in biological systems over time has been widely proposed as the primary contributor to the progressive deteriorative nature of aging [1–3]. The ever-increasing aging population will pose a significant challenge to the healthcare system as age is the biggest risk factor for chronic and complex conditions, such as neurodegenerative diseases, cancers, and cardiovascular diseases. However, aging is a multifaceted process that varies between individuals such that some people live longer in good health, while others experience various pathologies. Although the basis of healthy and pathological aging is not entirely understood, the rate of aging in terms of lifespan and healthspan may be controlled, to a certain extent, by interventions such as caloric restriction [4] and physical activity [5]. Aging is an inevitable process, but the identification of age-associated metabolites might be useful to understand the age-related changes of metabolisms in a healthy population and later compared with geriatric disorder-related metabolites to monitor the risk of developing age-associated disorders and help to provide novel insights into the underlying basis of aging.

Omics studies such as genomics, transcriptomics, proteomics, and metabolomics could be applied to elucidate the cellular signaling of a complex process. Being downstream of biochemical pathways, the metabolome has been regarded as the closest biomolecules to the phenotypes and physiological functions than other omics approaches. There has been a growing interest in metabolomics studies for aging research over the last decade due to advanced high-throughput analytical techniques using nuclear magnetic resonance spectroscopy and mass spectrometry [6]. Several cross-sectional and longitudinal studies have reported the metabolomics changes with age and gender using samples from different cohorts [7–19]. Although these studies provide essential information, the age-associated metabolome has not been fully characterized due to the diversity of metabolites. Besides the genetic determinants, the metabolome is influenced by environmental factors that could be highly dynamic across populations. Additional studies from different populations may reveal common metabolites associated with age regardless of sample origins and unique metabolite changes specific to a population.

To the best of our knowledge, the age-associated metabolome of Malays has not been reported in the literature. Our previous study shows that increased oxidative stress is associated with advanced age in Malays, such that DNA damage and protein carbonyl are predictors for cognitive impairment in the population [20]. Further study on the transcriptome of peripheral blood mononuclear cells (PBMCs) indicates that gene expressions related to inflammation, signal transduction, metabolic, and nociception pathways are altered with age in the Malays [21]. Our proteomics profiling of plasma from the Malays reports that the age-associated protein changes are related to the cholesterol metabolic process, fatty acid biosynthetic process, and response to toxic substances [22]. Metabolomics can complement other omics data by understanding the biochemical events in a biological system from a different perspective, such as the influence of dietary and environmental exposures, gut microbiota composition, and disease conditions. Integrating the information gathered from multiomics studies enables comprehensive evaluation of the molecular alterations during aging and facilitates the discovery of robust age-associated metabolites. Such metabolites may be used to understand the changes in metabolisms due to age and discern the risk of developing geriatric syndromes and diseases in a population. Therefore, this cross-sectional study is aimed at identifying the plasma age-associated metabolites for Malays.

## 2. Materials and Methods

### 2.1. Study Population

This study was approved by the Human Research Ethics Committee of the National University of Malaysia (UKM), which followed the Declaration of Helsinki (approval code: 1.5.3.5/244/LRGS/BU/2012/UKM_UKM/K04). All participants were provided the written informed consent to this study. The study was a cross-sectional study that recruited subjects in Klang Valley, Malaysia, from May 2013 to March 2015. The plasma samples were thawed in 2018 for LCMS analysis. The study design and methods have been described in detail elsewhere [20–23]. Briefly, participants selected for the metabolomics analysis consisted of healthy Malays based on self-reports who had no known physical or mental illness. The selected participants were of Malay race and free from any physical, health, and mental disorders at the time of recruitment. The exclusion criteria included subjects who were smokers, pregnant, diagnosed with diseases, and on medications for cardiovascular diseases, diabetes mellitus, hypertension, and cholesterol-lowering. The food and supplement intakes of the participants were not assessed.

### 2.2. Sample Collection

Overnight-fasted blood was collected at 8:00 a.m. to 10:30 a.m. The peripheral venous blood was drawn into an EDTA tube (Vacutainer, BD; Franklin Lakes, NJ, USA) and processed immediately after the collection. Briefly, the blood was centrifuged at 3,000 × g for 30 min at 4°C. The plasma was aliquoted and stored at -80°C until use.

### 2.3. Metabolite Extraction

Untargeted metabolomics analysis on plasma samples was performed using ultra-high-performance liquid chromatography-tandem mass spectrometry (UHPLC-MS/MS). Methanol, water, acetonitrile (ACN), and formic acid were MS grade and purchased from Fischer Scientific (Hampton, NH, USA). Metabolite extraction for the plasma samples was performed according to the previous protocol with slight modifications [24]. Briefly, 400 *μ*L plasma was added with 1,200 *μ*L cold methanol, vortex mixed for 15 s, and centrifuged at 15,800 × g for 15 min at 4°C. All the supernatant (~1.6 mL) was transferred to a new tube and dried using a vacuum centrifuge (Eppendorf, Hamburg, Germany) at room temperature. The dried samples were stored at -80°C until use.

Quality control (QC) samples were prepared by pooling an aliquot of 20 *μ*L from each plasma sample. The pooled QC samples were divided into several tubes, and metabolite extraction was carried out as described for the plasma sample. The samples were divided into 6 batches for data acquisition (18 for batch 1, 30 for batch 2, 31 for batches 3 and 4, 25 for batch 5, and the remaining for batch 6). Subjects were divided with an approximately equal representation of each age group for the 30s, 40s, 50s, and 60s.

### 2.4. LC-MS/MS

The dried samples were reconstituted with 200 *μ*L water, vortexed for 15 s, and spun down quickly. The samples were filtered through a 0.2 *μ*m GHP membrane (Waters; Milford, MA, USA) and transferred into the vial with a glass insert (Thermo Scientific; Waltham, MA, USA). Water was used as the blank sample. Data were acquired using Q Exactive HF Orbitrap (Thermo Scientific) according to a previous study with slight modification [24]. The detailed protocol is included in Supplementary Information Method 1.

### 2.5. Data Preprocessing

Raw files generated from LCMS were processed using Compound Discoverer software (version 2.0; Thermo Scientific). Briefly, chromatographic peaks were aligned with other files within a mass tolerance of 5 parts per million (ppm) and grouped by molecular weight and retention time. Background compounds were filtered out when the sample/blank (S/N) signal ratio was <5. The minimum intensity for a peak to be reported was defined as 2,000,000. The area of missing peak for a group compound was imputed using the “Fill Gap” node in Compound Discoverer, defined as “expected peak width and maximum spectrum noise in the expected retention time range multiplied by the S/N threshold” based on the software user guide. The metabolite features (MFs) with molecular weight, retention time, and peak intensity information were exported into a comma-separated values (CSV) file for further analysis.

### 2.6. Statistical Analysis

Statistical analysis was first performed on MetaboAnalyst 4.0 [25]. Briefly, the data in the form of an MS peak list was uploaded as a CSV file. Peaks were grouped at a mass tolerance of 0.025 *m*/*z* and retention time tolerance of 30 s. Data were filtered using the interquartile range, while the normalization was performed using cube root transformation and autoscaling. Principal Component Analysis (PCA) was used to assess the batch effects of individual and combined runs. The batch effects of the combined runs were corrected using the ComBat method [26] available in MetaboAnalyst. The batch effect-corrected data were used for further analysis.

The general linear model was used for the association analysis (IBM SPSS, v22; Armonk, NY, USA). The model on gender-associated metabolites was adjusted for age. The effect size (Cohen's *d*) was calculated based on the standardized difference between the two means. Metabolites associated with age were adjusted for gender. Gender-stratified analysis was also performed to assess the association of metabolites with age in males and females, respectively. False discovery rate-adjusted *p* value (*q*-value as determined by the Benjamini-Hochberg procedure) < 0.05 was considered statistically significant. Additional filtering based on the coefficient of variation (CV) of QC was applied to the significantly different metabolites such that metabolites with values ≤ 30% were considered acceptable.

### 2.7. Metabolite Annotation

The significant MFs were putatively annotated by matching their accurate mass and MS/MS spectrum to the online databases. Briefly, the mzCloud database (HighChem LLC, Slovakia) has been integrated into Compound Discoverer, and annotation was performed automatically. Then, accurate masses of the remaining unannotated significant MFs were first searched against CEU Mass Mediator version 3.0 [27], which unified databases from METLIN [28] and Human Metabolome Database (HMDB) [29]. The mass tolerance was set at 5 ppm. The MFs that matched with CEU Mass Mediator were searched against the MS/MS databases of METLIN and HMDB with a mass tolerance of 5 ppm and MS/MS tolerance of 0.01 Dalton.

The level of confidence annotation was based on the recommendation from Metabolomics Standard Initiative [30]. Only significant MFs with level 2 confident annotation were reported as putative metabolites. Level 2 confident annotation was defined in this study as the compound had matched two orthogonal parameters: accurate mass (<5 ppm) and MS/MS spectrum (≥70% similarity compared to the database). The mzCloud Best Match score (%), HMDB purity (%), and METLIN score (%) were generated automatically by the respective databases.

## 3. Results

### 3.1. Characteristics of the Participants

A total of 146 individuals were included in this study (Table 1). Of these, 90 (61.6%) and 56 (38.4%) were females and males, respectively. The participants were aged from 28 to 69 years old.

### 3.2. Data Integrity

**Figure 1:**
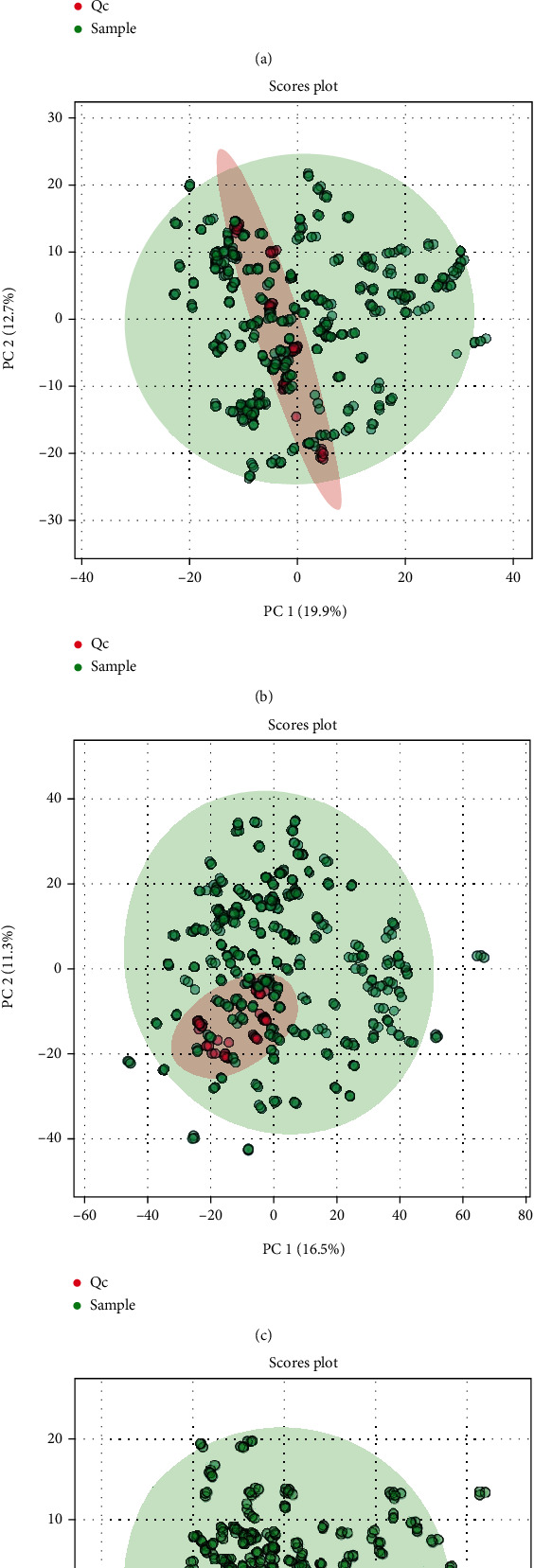
PCA score plot of sample and QC combined from all runs. (a) Positive and (b) negative ion modes before batch effect correction. (c) Positive and (d) negative ion modes after batch effect correction.

Mass spectrometry data were acquired in 6 runs (Supplementary Figures S1 and S2). The integrity of the combined data from 6 runs was evaluated based on the distribution of the QC samples in the score plot of PCA. The QC samples from the combined runs were scattered in the score plots for both ion modes (Figure 1). These findings indicate that batch effects existed among different runs. After batch effect correction, the QC samples were more closely clustered together. A total of 2,424 and 834 MFs were detected in positive and negative modes, respectively (Supplementary Table S1).

### 3.3. Gender-Associated Metabolites

Levels of 18 metabolites were different between males and females after adjusting for age (Table 2). The majority (17/18) of these metabolites had higher levels in males than females. 4-Hydroxyphenyllactic acid (4HPLA), indole-3-lactic acid, N-acetyl-L-carnosine, testosterone sulfate, 3-phenyllactic acid, cortisol, propionylcarnitine, and carnitine were among the metabolites with the effect size > 0.4, and their levels were higher in males than females. Oleamide was the only metabolite identified to be higher in females than males.

### 3.4. Age-Associated Metabolites

Metabolites were assessed by association with age after being adjusted for gender or in gender-stratified analysis. Regression analysis showed that 1020/2424 (42.1%) and 325/834 (39%) of MFs were associated with age (Supplementary Table S1). Out of these, only 58 metabolites were identified as level 2 confident annotation. Furthermore, only 26/58 (44.8%) of these metabolites had QC CV ≤ 30%. Metabolites that were positively associated with age included 3-methoxybenzenepropanoic acid, 4-oxoproline, deoxycholic acid, glutamine, hippuric acid, N-acetylornithine, and N-phenylacetylglutamine, while those negatively associated with age were 11-deoxy prostaglandin F2*β*, 9(E),11(E)-conjugated linoleic acid, cortisol, guanosine monophosphate, N-acetyl-L-carnosine, nicotinamide, phenylalanine, proline, testosterone sulfate, and uracil (Table 3).

In gender-stratified analysis, only 40 and 36 age-associated metabolites in males and females, respectively, were annotated confidently at level 2. Out of these, 20/40 (50%) and 16/36 (44.4%) metabolites had QC CV ≤ 30%. In males, metabolites that had a direct association with age included 3-methoxybenzenepropanoic acid, deoxycholic acid, docosahexaenoic acid ethyl ester (DHAEE), glutamine, N-acetylornithine, and N-phenylacetylglutamine, while those that had an inverse association with age were 11-deoxy prostaglandin F2*β*, 13,14-dihydro-15-keto prostaglandin A2, 9(E),11(E)-conjugated linoleic acid, guanosine monophosphate, nicotinamide, proline, testosterone sulfate, and uracil (Table 3). In females, metabolites such as 4-oxoproline, glutamine, and N-acetylornithine were directly associated with age, while metabolites like 11-deoxy prostaglandin F2*β*, cortisol, glycoursodeoxycholic acid, guanosine monophosphate, testosterone sulfate, and tryptophan were inversely associated with age (Table 3).

## 4. Discussion

The discovery of age-associated metabolites could be useful in understanding the metabolic alteration due to aging and stratifying populations that will undergo healthy aging or are at risk of developing age-related disorders. Metabolites have the potential to be used as biomarkers in the clinical setting because they are closely related to the physiological and health status of an individual. In addition, they are relatively robust to be developed as a panel of markers that allows rapid screening or as easy-to-use devices to monitor personal health status without involving the use of sophisticated instruments in the laboratory. In this study, we determined the plasma metabolome of the apparently healthy Malays based on a self-reported questionnaire to identify the circulating metabolites associated with age and gender. We observed several metabolites that showed variation between genders or changed with age in this population, and some of these have been reported in other populations. We performed the pathway analysis using MetaboAnalyst (data not shown) but only 1 pathway (arginine biosynthesis) was significantly different (FRD < 0.05) and impactful (>0.1). This is probably because most of the differentially expressed metabolites are not related to each other in the pathway analysis. Therefore, changes in individual metabolites in relation to age and sex were discussed.

For gender-specific metabolites, we observed that a higher level of carnitine in males has been reported by several studies [8, 10, 15, 18], but the reason for the gender difference remains unclear. Of note, 4HPLA had the largest effect size (*d* = 0.90). This is consistent with a previous study that shows that 4HPLA level is higher in males than females, possibly related to alcohol consumption or gut microbial cometabolism [14]. As this population was nondrinker, there might be a gender-specific difference in the interplay between the gut microbiome and host metabolisms which warrants further investigation. In addition, 4HPLA is one of the metabolites in tyrosine metabolism. A higher level of 4HPLA in the males of this study may imply an increased consumption of protein-rich foods. The claim is corroborated by a meta-analysis that found higher protein intake in Malaysian males than females [31]. However, the inference remains to be verified as dietary patterns were not examined in the current study.

DHAEE was found to be gender- and age-related in this study. It is a stabilized metabolite from the supplementation of *ω*-3-acid ethyl esters [32]. Males in this study had a higher level of DHAEE than females, suggesting a gender difference in the clearance of the compound. The DHAEE level was directly associated with age in Malay males. In addition, 9(E),11(E)-conjugated linoleic acid, a form of *ω*-6 fatty acid, was inversely associated with age in Malay males. Interestingly, our previous transcriptomics study shows that endogenous synthesis of *ω*-3 and *ω*-6 fatty acids is impaired in the older Malays due to downregulation of FADS genes that are involved in the conversion of dietary *α*-linolenic acid and linoleic acid to *ω*-3 and *ω*-6 fatty acids, respectively, supporting the recommendation of long-chain fatty acid supplementation based on FADS gene expressions [21].

In this study, levels of 13,14-dihydro-15-keto prostaglandin A2 and 11-deoxy prostaglandin F2*β* had a negative association with age in males and both genders, respectively. Prostaglandins are divided into many types and perform diverse biological functions. Prostaglandin E_2_ (PGE_2_) is commonly linked to the generation of an inflammatory response [33], and its production is inhibited by DHA *in vitro* [34]. In addition, DHA has been shown to decrease the production of PGE_2_ in human umbilical vein endothelial cells [35]. Supplementing fish oil, which contains eicosapentaenoic acid and DHA, decreases the production of PGE_2_ [36]. Dietary supplementation of DHA-rich fish oil has been found to reduce the release of PGF_2*α*_ from the lipopolysaccharide-stimulated PBMCs derived from Alzheimer disease patients in the OmegAD study [37]. In addition, fish oil supplementation has been correlated with a reduction of serum PGE_2_ levels in patients with nonalcoholic fatty liver disease associated with hyperlipidemia [38]. Nevertheless, the functions of prostaglandins detected in this study are not well described in the literature, and their levels could be influenced by other factors such as cellular damage, inflammation, infection, and age-related conditions. The roles of the 13,14-dihydro-15-keto prostaglandin A2 and 11-deoxy prostaglandin F2*β* in aging and their potential relationship with the long-chain fatty acid intake remain to be investigated.

Tryptophan is an essential amino acid that is catabolized mainly through the kynurenine pathway to produce kynurenine and other downstream metabolites linked to aging and age-related diseases [39]. In a human study, lower plasma tryptophan level is associated with decreased olfactory function in healthy elderly [40]. The elderly with mood disorders have a lower level of urinary tryptophan, possibly related to lower daily intake of tryptophan and increased tryptophan degradation via the kynurenine pathway [41]. Supplementing a tryptophan-enriched diet improves the recognition of positive emotions in the healthy elderly [42]. These findings suggest that tryptophan is directly associated with healthy aging, and supplementation has the potential to ameliorate geriatric disorders elicited by tryptophan deficiency. A previous study on the Malaysian elderly reports that urinary tryptophan level is higher in the elderly with intact cognition than those with mild cognitive impairment (MCI) [43], most probably due to higher intake of dairy products which are rich in tryptophan by the former group [44]. The apparently healthy elder Malays, especially the females, might be at higher risk of developing cognitive decline due to a lower level of tryptophan. The gender-specific association could be attributed to an accelerated rate of tryptophan degradation in older females than males [45].

The kynurenine/tryptophan ratio in the blood is increased with age and has been proposed as a biomarker for inflammaging due to parallel activation of inflammation mediators [46]. Increased kynurenine/tryptophan ratio is related to frailty [47], risk of developing cardiovascular disease [48], cognitive decline [49], and mortality [50] in older adults. Interestingly, we observed that circulating kynurenine level was detected but not affected with age in this study, possibly due to compartmental transport of tryptophan and kynurenine such that degradation of systemic tryptophan increases intracellular kynurenine [46]. Therefore, the plasma kynurenine/tryptophan ratio was considered as increased with age in this study. As our body cannot produce tryptophan, supplementation of tryptophan could be beneficial to the elder Malays. This is supported by a previous study which indicates that successful-aged Malaysians are associated with higher urinary tryptophan level [43].

Hippuric acid is a gut microbiota-derived metabolite from the degradation of dietary polyphenols available in vegetables, fruits, coffee, and tea [51]. Its level reflects the change in the dietary pattern or gut microbiota composition. Interestingly, the hippuric acid level increases with age but decreases in age-related conditions. A longitudinal study of an Italian population shows that a high level of plasma hippuric acid is associated with a reduced risk of developing frailty, and its elevated level is linked to increased fruit–vegetable intake [52]. Another study on the Japanese population reports that a low level of whole-blood hippuric acid in the elderly is related to hypomobility, while a low tryptophan level is linked to frailty and cognitive impairment [53]. A previous study reports that successful-aged Malaysians without MCI are associated with the intake of oats and tropical fruits [44]. We observed that the plasma level of hippuric acid was positively associated with age in this population. Further studies are required to associate the level of hippuric acid with polyphenol consumption in the elderly.

We observed a change in glutamine level with age. Glutamine is the most abundant free amino acid in the body. Skeletal muscle is the primary tissue supply for glutamine in the bloodstream [54]. Circulating glutamine levels can be varied by exercise, fasting, and injury. Prolonged and strenuous exercise reduced circulating glutamine levels, possibly attributed to the enhanced uptake by the liver and other tissues and the reduced glutamine synthesis in the muscle [55]. A low level of circulating glutamine may reflect muscle loss, but glutamine supplementation does not seem to prevent sarcopenia [56]. Our data showed that glutamine level was positively associated with age. Further studies can be performed to determine the association between plasma glutamine levels and muscle loss.

Cholesterol is the precursor for synthesizing compounds such as bile acids and steroid hormones. Cholesterol was not identified in this study, but its derivatives, including cortisol, testosterone sulfate, and bile acids, were associated with age or gender. We observed that cortisol was related to gender and age. Its level was higher in males than females and inversely associated with age in females. These findings differ from previous studies on gender differences [57] and females during aging [8, 58]. The inconsistencies might be due to sample types, as the earlier study measured the free cortisol in saliva [57], while our study could not discern the free form from total cortisol in plasma. In addition, cortisol level is inversely associated with the waist-hip ratio in females [57], and the age-associated metabolites were adjusted with body mass index (BMI) [8]. In contrast, anthropometric measurements were not assessed in this study but the participants were standardized that they were free from metabolic diseases and syndromes. As various conditions, including adiposity as a confounding factor, could trigger cortisol release, the possible explanations for the contradictory findings reported in different populations remain to be investigated. The level of testosterone sulfate, a conjugated form of testosterone, was negatively associated with age in Malay males and females. The findings could be attributed to decreased testosterone production during aging [59]. In addition, testosterone sulfate level was higher in Malay males than females, which is likely due to testosterone being the primary sex hormone in males. In this study, the deoxycholic acid level was associated with increased age in males, while glycoursodeoxycholic acid level was higher in males than females and inversely associated with age in females. Other studies have also reported that glycoursodeoxycholic acid level is higher in males, but the association patterns of these bile acids with age are inconsistent with our data [60, 61]. Bile acid profile can be influenced by BMI [61] and body fat percentage but is not associated with total fat intake [60]. In addition, intestinal bacteria produce deoxycholic acid and glycoursodeoxycholic acid. The roles of gut microbiota on the secondary bile acid production during aging remain to be confirmed as one of the possible modifiers. The previous study has shown that lipid profile, especially the lipoprotein metabolism, is altered in elder Chinese Singaporean, which could be an indicator of aging [62]. Our previous transcriptomics and proteomics studies show that several expressions of genes and proteins related to cholesterol metabolism are altered with age in Malays. These include age-associated increased expression of the TM7SF2 gene, which is involved in cholesterol biosynthesis, and decreased ALOX5 expression, a gene taking part in leukotriene production [21]. At the protein level, lipoprotein AOPA4 is higher in Malays beyond 60 years, while lipoprotein (a) decreases with age [22]. These findings indicate the complicated modulation of cholesterol metabolism which is altered at different molecular levels during aging. A lipidomics study is warranted to comprehensively characterize the lipid changes. The altered pathways might be related to factors such as anthropometric variation and gut microbiota composition which shall be considered in future studies.

In addition, the age-associated metabolites in this study were compared with previous studies [7–14, 16, 17, 19] to identify potential common age-associated metabolites across different populations. Levels of aconitic acid [8, 9], N-phenylacetylglutamine [8, 10], glutamine [8–10], and hippuric acid [8–10] are associated with increased age as reported by this and other studies. On the other hand, levels of proline [7, 8] and nicotinamide [8, 19] are inversely associated with age in the males of this and other studies. Glutamine level is associated with increased age in the females of this and other studies [8, 14]. Besides, the level of tryptophan [7, 8, 12, 16] is lower with age in the females of this and other studies. However, compared to this study, phenylalanine level with age [8–10] and glutamine level with age in males [14, 16] are opposite to other studies. Taken together, these findings suggest that several age-associated metabolites are consistently observed across different populations, suggesting that their metabolisms are altered during aging regardless of population origins. On the other hand, some metabolites might be varied in different populations due to several factors. The variations could be attributed to technical reasons such as interlaboratory and methodology variabilities or biological factors such as genetic backgrounds and lifestyles. The differences include the previous study populations that were aged 18 to 100 years old from Europe [7, 10, 12, 16], US [8, 9], and East Asia [11, 17, 19]; and some studies were longitudinal [7, 8], which might explain the part of the inconsistencies between studies. Notwithstanding, US-based population studies show that age-associated metabolites are only moderately correlated with metabolite heritability [8] and are not affected by race [9]. Adiposity could influence the levels of metabolites [9, 14, 16], but participants' BMI, dietary intake, and physical activity were not assessed in this study. Although BMI is not directly correlated with body fatness [14], it is a relatively easy surrogate measure for adiposity. In addition, the inconsistencies between findings might be related to factors specific to the study population, including dietary habits, lifestyle, and gut microbiota composition. Some of these metabolites are associated with age independent of gender, while others are associated with age in either gender only. The association of metabolites with age in a gender-dependent manner could be attributed to the effect of age-gender interaction, which could be assessed in future studies.

The strength of our study is the identification of a panel of age-associated metabolites in the Malay population which can be used as a reference dataset for future studies to assess healthy aging and age-related disorders. These age-associated metabolites could reflect the dietary intake, gut microbiota composition, and physical activity of the participants. The alteration of metabolic pathways in the elder Malays might be used as an indication to monitor the predisposed risk of developing age-related disorders. Further studies on dietary patterns, physical activity, gut microbiota composition, and anthropometric measurements are required to confirm the mechanistic insights of age-associated metabolites. These metabolites shall be correlated further with geriatric morbidities, frailty, and physiological functions to find objective biomarkers that can discern healthy aging from pathological ones and be used to develop appropriate recommendations for the early prevention of age-related diseases.

The limitation of this study is that the metabolite levels were not adjusted with other confounding factors, including BMI, systolic blood pressure, dietary intake, physical activity, conditions (fasting and exercise) before blood collection, and lifestyle as reported by previous studies [7, 8, 10, 14, 16]. We could not evaluate the contribution of these factors to age-associated metabolites. Nevertheless, the subjects were standardized to be free from metabolic diseases and syndromes. Second, the participants were recruited in the central region of Peninsular Malaysia, which might limit the generalization to other populations. Cohort studies with a longitudinal design, larger sample size, and broader area coverage that consider the covariables are needed to understand the roles of age-associated metabolites in mediating the functional outcomes of the elderly population. Lastly, absolute quantification of the age-associated metabolites was not performed to enable comparison with other studies. Targeted and lipidomics profiling is recommended to confirm the altered metabolic pathways during aging.

## 5. Conclusions

In conclusion, this study reveals several age-associated metabolites in Malays such as DHAEE, 9(E),11(E)-conjugated linoleic acid, nicotinamide, tryptophan, glutamine, hippuric acid, cortisol, testosterone sulfate, deoxycholic acid, and glycoursodeoxycholic acid. These age-associated metabolites reflect the changes in metabolisms during aging in the Malays and could be used to discern the risk of developing age-related diseases later. Further studies are warranted to determine the influence of factors such as genetic background, lifestyle, gut microbiota composition, physical activity, and dietary intake on the altered metabolic processes during aging and relate the metabolites to functional outcomes.

## Figures and Tables

**Table 1:** Demographic data of the participants.

Characteristic	Total	Males	Females
Number of participants	146	56 (38.4%)	90 (61.6%)
Age range in years	28 - 69	28 - 69	28 - 65
Mean age in years (SD)	44.3 (11.6)	47.5 (12.2)	42.3 (10.7)

**Table 2:** Metabolites associated with gender.

Compound name	Ionization mode	Signal level in males (SD)^∗^	Signal level in females (SD)^∗^	Mean difference (m-f)^∗^	Effect size (*d*)	*p* value
4-Hydroxyphenyllactic acid (4HPLA)	—	3.3*e*5 (1.39*e*5)	2.0*e*5 (1.38*e*5)	1.2*e*5	0.90	7.1*e*-18
Indole-3-lactic acid	—	2.5*e*5 (8.60*e*4)	1.8*e*5 (8.55*e*4)	7.4*e*4	0.87	4.1*e*-17
N-Acetyl-L-carnosine	+	3.1*e*4 (2.25*e*4)	1.3*e*4 (2.24*e*4)	1.7*e*4	0.78	4.7*e*-14
Testosterone sulfate	—	6.0*e*6 (2.82*e*6)	4.0*e*6 (2.81*e*6)	2.0*e*6	0.71	4.9*e*-12
3-Phenyllactic acid	—	8.8*e*4 (4.11*e*4)	6.0*e*4 (4.08*e*4)	2.8*e*4	0.68	2.7*e*-11
Cortisol	+	8.6*e*4 (3.54*e*4)	7.0*e*4 (3.52*e*4)	1.64*e*	0.46	5.0*e*-6
Propionylcarnitine	+	5.5*e*5 (1.94*e*5)	4.6*e*5 (1.93*e*5)	8.3*e*4	0.43	1.8-*e*-5
Carnitine	+	3.9*e*7 (9.54*e*6)	3.5*e*7 (9.49*e*6)	4.0*e*6	0.43	2.4*e*-5
Oleamide	+	2.5*e*6 (2.58*e*6)	3.5*e*6 (2.56*e*6)	-1.0*e*6	-0.40	7.0*e*-5
Docosahexaenoic acid ethyl ester (DHAEE)	+	3.3*e*5 (2.82*e*5)	2.2*e*5 (2.81*e*5)	1.1*e*5	0.39	9.6*e*-5
Glycoursodeoxycholic acid	—	1.7*e*6 (1.40*e*6)	1.2*e*6 (1.39*e*6)	5.5*e*5	0.39	9.7*e*-5
Gamma-Glu-Leu	—	3.7*e*5 (1.94*e*5)	2.9*e*5 (1.93*e*5)	7.5*e*4	0.39	1.0*e*-4
Theophylline	—	2.2*e*5 (1.82*e*5)	1.6*e*5 (1.81*e*5)	6.3*e*4	0.35	5.2*e*-4
Hippuric acid	+	1.6*e*5 (1.39*e*5)	1.2*e*5 (1.38*e*5)	4.2*e*4	0.31	2.0*e*-3
Proline	+	1.2*e*7 (4.15*e*6)	1.1*e*7 (4.13*e*6)	1.3*e*6	0.31	2.0*e*-3
Indole-3-acetic acid	+	2.5*e*5 (8.60*e*4)	1.8*e*5 (8.55*e*4)	2.3*e*4	0.31	2.1*e*-3
Xanthine	—	3.2*e*5 (1.40*e*5)	2.8*e*5 (1.39*e*5)	4.1*e*4	0.30	3.1*e*-3
N-Acetylornithine	+	1.7*e*5 (7.54*e*4)	1.4*e*5 (7.50*e*4)	2.2*e*4	0.29	3.4*e*-3

^∗^Signal intensity adjusted for age (arbitrary unit); f: females; m: males; SD; standard deviation; positive value represents higher level in males, negative value represents higher level in females; compounds were sorted according to effect size.

**Table 3:** Metabolites associated with age.

Compound name	Age^a^		Males^b^		Females^c^	
	*β*	*p* value	*β*	*p* value	*β*	*p* value
Positively associated with age						
3-Methoxybenzenepropanoic acid	0.32	2.2*e*-11	0.27	3.8*e*-4	0.35	6.1*e*-9
4-Methylphenol	—	—	0.26	6.2*e*-4	—	—
4-Oxoproline	0.16	8.0*e*-4	—	—	0.22	3.4*e*-4
Aconitic acid (cis/trans)	0.14	3.7*e*-3	—	—	—	—
Caprolactam	0.14	3.4*e*-3	—	—	0.25	3.6*e*-5
Citrulline^∗^	0.25	2.7*e*-7	0.24	2.1*e*-3	0.25	3.6*e*-5
Deoxycholic acid	0.12	1.7*e*-2	0.29	1.1*e*-4	—	—
Docosahexaenoic acid ethyl ester (DHAEE)	—	—	0.28	2.5*e*-4	—	—
Erucamide	0.32	1.2*e*-11	0.22	3.9*e*-3	0.38	7.4*e*-11
Glutamine^∗^	0.19	9.9*e*-5	0.23	2.3*e*-3	0.16	8.1*e*-3
Hippuric acid^∗^	0.17	4.8*e*-4	—	—	—	—
N-Acetylornithine	0.27	2.2*e*-8	0.20	9.1*e*-3	0.31	1.6*e*-7
N-Phenylacetylglutamine^∗^	0.23	2.0*e*-6	0.36	2.0*e*-6	—	—
Pro Phe Arg	0.15	2.4*e*-3	—	—	0.20	9.1*e*-4
Theophylline	0.15	2.4*e*-3	0.19	1.2*e*-2	—	—
Negatively associated with age						
11-Deoxy prostaglandin F2*β*	-0.21	4.4*e*-5	-0.27	6.9*e*-4	-0.16	1.0*e*-2
12-Hydroxy-10-(hydroxymethyl)-6-methyl-2-(4-methyl-3-penten-1-yl)-2,6,10-dodecatrienoic acid	-0.19	8.6*e*-5	-0.25	9.4*e*-4	-0.16	1.0*e*-2
13,14-Dihydro-15-keto prostaglandin A2	—	—	-0.21	6.7*e*-3	—	—
9(E),11(E)-Conjugated linoleic acid	-0.19	7.1*e*-5	-0.28	2.6*e*-4	—	—
Cortisol	-0.20	3.6*e*-5	—	—	-0.23	1.1*e*-4
Glycoursodeoxycholic acid	—	—	—	—	-0.18	3.1*e*-3
Guanosine monophosphate	-0.24	9.1*e*-7	-0.32	2.2*e*-5	-0.17	4.1*e*-3
Isoquinoline	-0.12	1.5*e*-2	—	—	—	—
Leu Tyr	-0.21	2.1*e*-5	-0.29	1.6*e*-4	-0.15	1.2*e*-2
N-Acetyl-L-carnosine	-0.11	1.8*e*-2	—	—	—	—
Nicotinamide	-0.20	3.9*e*-5	-0.28	2.2*e*-4	—	—
Phenylalanine	-0.12	1.5*e*-2	—	—	—	—
Proline	-0.14	4.0*e*-3	-0.24	1.9*e*-3	—	—
Testosterone sulfate	-0.57	1.3*e*-38	-0.60	6.3*e*-18	-0.54	6.6*e*-22
Tryptophan	—	—	—	—	-0.15	1.3*e*-2
Uracil	-0.20	3.3*e*-5	-0.37	1.0*e*-6	—	—

^a^Age-associated metabolites adjusted for gender; *β*: unstandardized coefficients; ^b^age-associated metabolites in males; ^c^age-associated metabolites in females; ^∗^detected in both modes, values shown for positive mode; compounds were sorted according to alphanumerical order.

## Data Availability

Data is available on request.

## Supplementary Materials

Supplementary Materials Supplementary Information Method 1: LCMS method. Supplementary Figure S1: PCA score plot of samples in runs 1–6 for positive ion mode. Supplementary Figure S2: PCA score plot of samples in runs 1–6 for negative ion mode. Supplementary Table S1: number of total MFs detected, significant MFs, and significant metabolites with level 2 confidence annotation.
