# Supplementary material for: Metabolomics Profiling of Age-Associated Metabolites in Malay Population
Source: Oxid Med Cell Longev. 2023 Feb 4;2023:4416410. doi: 10.1155/2023/4416410 (PMC9922189; doi:10.1155/2023/4416410)
Supplement: Supplementary Materials — Supplementary Information Method 1: LCMS method. Supplementary Figure S1: PCA score plot of samples in runs 1–6 for positive ion mode. Supplementary Figure S2: PCA score plot of samples in runs 1–6 for negative ion mode. Supplementary Table S1: number of total MFs detected, significant MFs, and significant metabolites with level 2 confidence annotation. [file 4416410.f1.zip › Supplementary Information Method 1.docx]

## Supplementary Description

**Supplementary Information Method 1**

LC-MS/MS

LC was performed on a UHPLC system (Dionex UltiMate 3000; Thermo Scientific) coupled to an Orbitrap MS/MS (Q Exactive HF; Thermo Scientific) through a heated electrospray ionization (ESI) probe. The instrument was calibrated with Pierce LTQ ESI Positive and Negative Ion Calibration Solutions (Thermo Scientific). LC separation was achieved by a C18 column (1.7 μm particle size x 100 mm length x 2.1 mm diameter; Synchronis, Thermo Scientific).

LC condition was set as followed: column temperature at 55 °C, autosampler temperature at 10 °C, injection volume at 2 μL, and flow rate at 0.45 mL/min. Solvent A was water and solvent B was ACN, each with 0.1 % formic acid (v/v). A 22 min elution gradient was set as followed: 0 min, 0.5 % B; 1 min, 0.5 % B; 16 min, 99.5% B; 20 min, 99.5% B; and 22 min, 0.5 % B.

The ESI was run on both positive and negative modes, separately. The ESI MS source was tuned as followed: sweep gas flow rate was adjusted to 50 arbitrary unit (AU), auxiliary gas flow rate at 18 AU, sweep gas flow rate at 0 AU, capillary temperature at 320 °C, S-lens level at 55 AU, auxiliary gas heater temperature at 300 °C, and spray voltage at 3.5 kV for positive mode or 3.0 kV for negative mode.

MS spectra were acquired thrice using full MS scan followed by data-dependent tandem mass spectrometry (ddMS2) scanning method as provided by Xcalibur 4.0 software (Thermo Scientific). Full MS scan was collected at a resolution of 60,000 and scan range of 100-1,000 mass/charge (m/z), while the ddMS2 scan was collected at a resolution of 15,000 and stepped normalized collision energy of 20, 40, and 60 AU.

Data acquisition for all samples was unable to be completed in a single run due to the large number of injections. The samples were divided into 6 runs. For each run, after completion of the positive mode acquisition, it was followed by negative mode acquisition. The QC and plasma samples were arranged according to previous study with slight modification (1). Briefly, the QC sample was injected at the beginning, throughout the data acquisition at every four samples, and the end of each run. The plasma samples were randomly arranged in between the QC injections. Each plasma sample was consecutively injected for three times as technical replicates.

**Reference:**

(1) Dunn WB, Broadhurst D, Begley P, Zelena E, Francis-Mcintyre S, Anderson N, Brown M, Knowles JD, Halsall A, Haselden JN, et al. Procedures for large-scale metabolic profiling of serum and plasma using gas chromatography and liquid chromatography coupled to mass spectrometry. Nat Protoc (2011) 6:1060–1083. doi:10.1038/nprot.2011.335
