# Supplementary material for: Metabolomics Profiling of Age-Associated Metabolites in Malay Population
Source: Oxid Med Cell Longev. 2023 Feb 4;2023:4416410. doi: 10.1155/2023/4416410 (PMC9922189; doi:10.1155/2023/4416410)
Supplement: Supplementary Materials — Supplementary Information Method 1: LCMS method. Supplementary Figure S1: PCA score plot of samples in runs 1–6 for positive ion mode. Supplementary Figure S2: PCA score plot of samples in runs 1–6 for negative ion mode. Supplementary Table S1: number of total MFs detected, significant MFs, and significant metabolites with level 2 confidence annotation. [file 4416410.f1.zip › Supplementary Materials_v2.pdf]

Supplementary Information

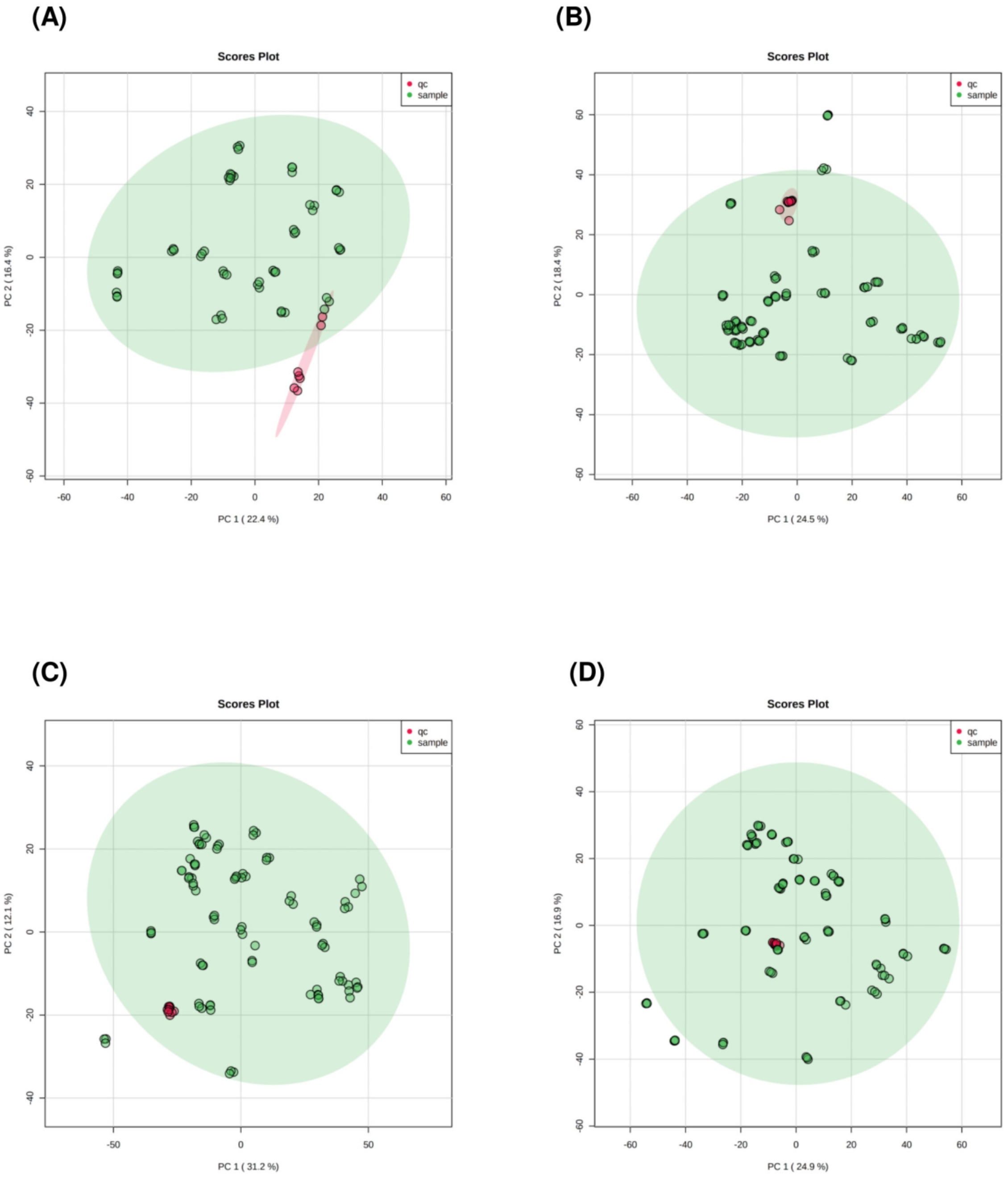

(E) (F)

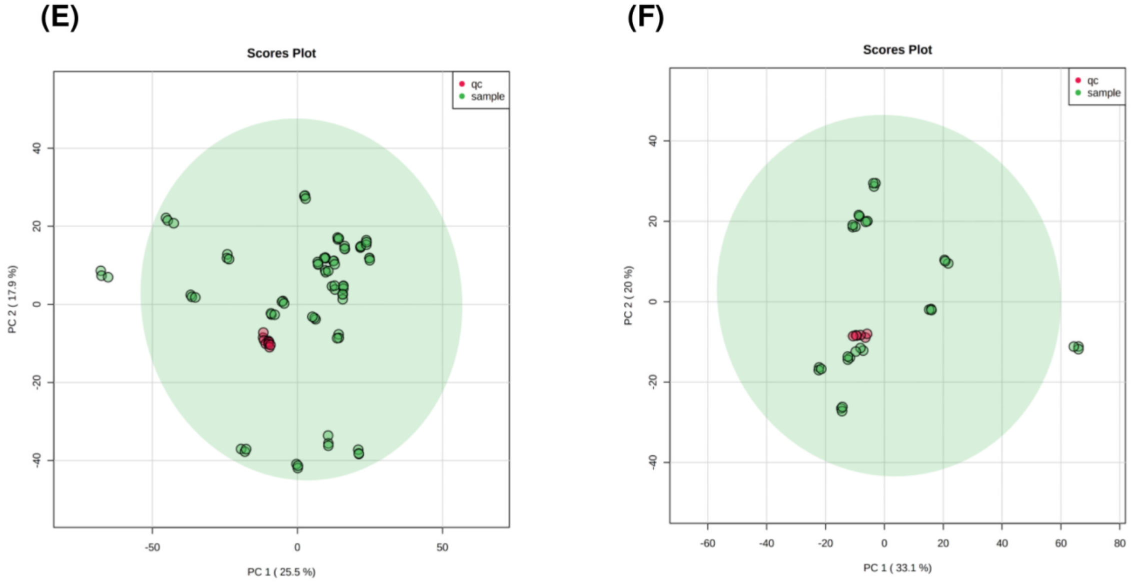

Figure S1. PCA score plot of samples in run 1 – 6 for positive ion mode. Run 1 to 6 (A to F). The QC samples in run 1 were scattered into 2 regions, implying existence of batch effects. QC samples of other runs were tightly clustered together within the respective runs, implying the runs were stable and reliable. Red dot is QC sample; green dot is plasma sample; shade area is the 95% confidence region.

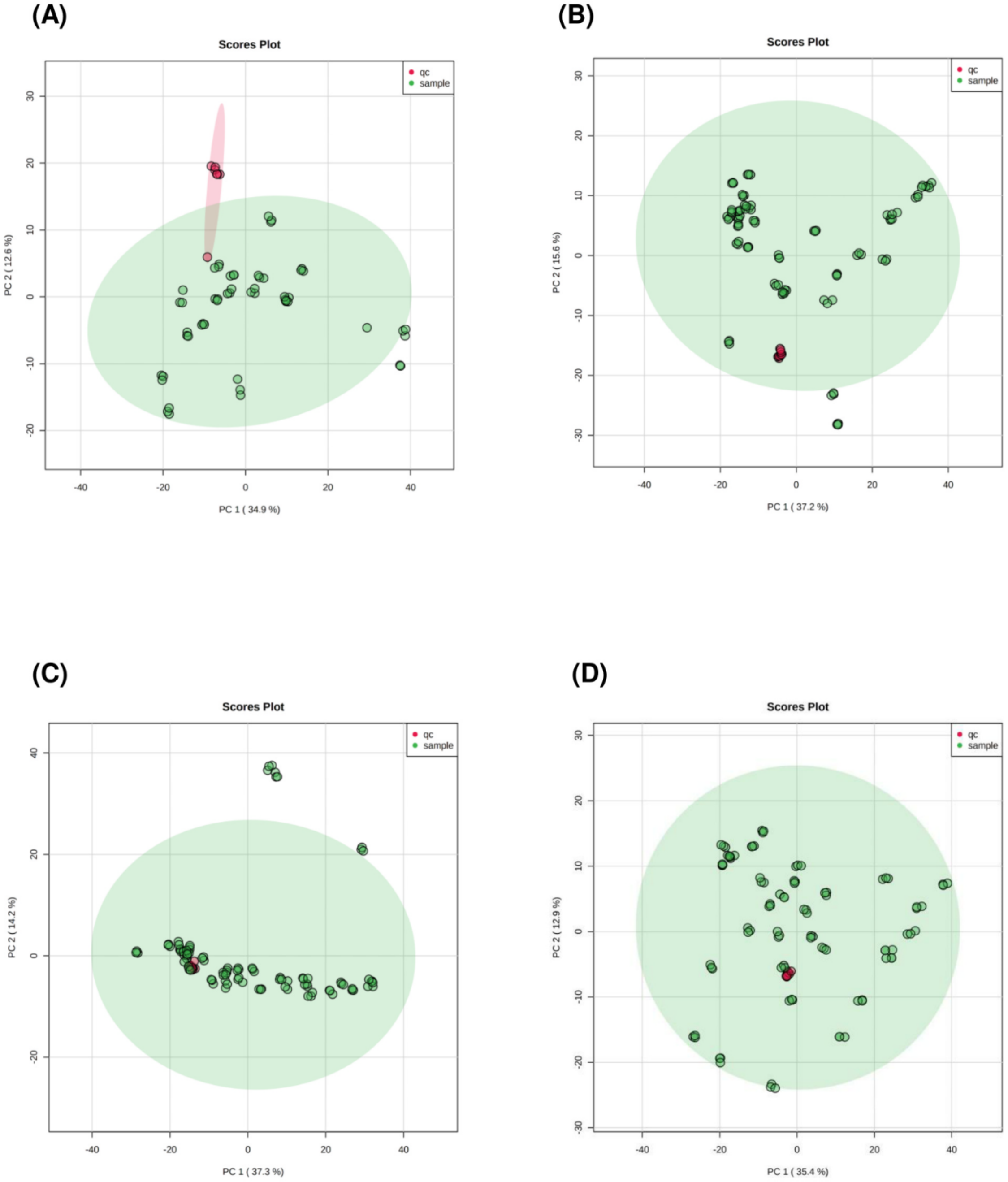
(A)
Scores Plot
qc
sample
PC 1 (34.9 %)
PC 2 (12.6 %)
(B)
Scores Plot
qc
sample
PC 1 (37.2 %)
PC 2 (15.6 %)
(C)
Scores Plot
qc
sample
PC 1 (37.3 %)
PC 2 (14.2 %)
(D)
Scores Plot
qc
sample
PC 1 (35.4 %)
PC 2 (12.9 %)

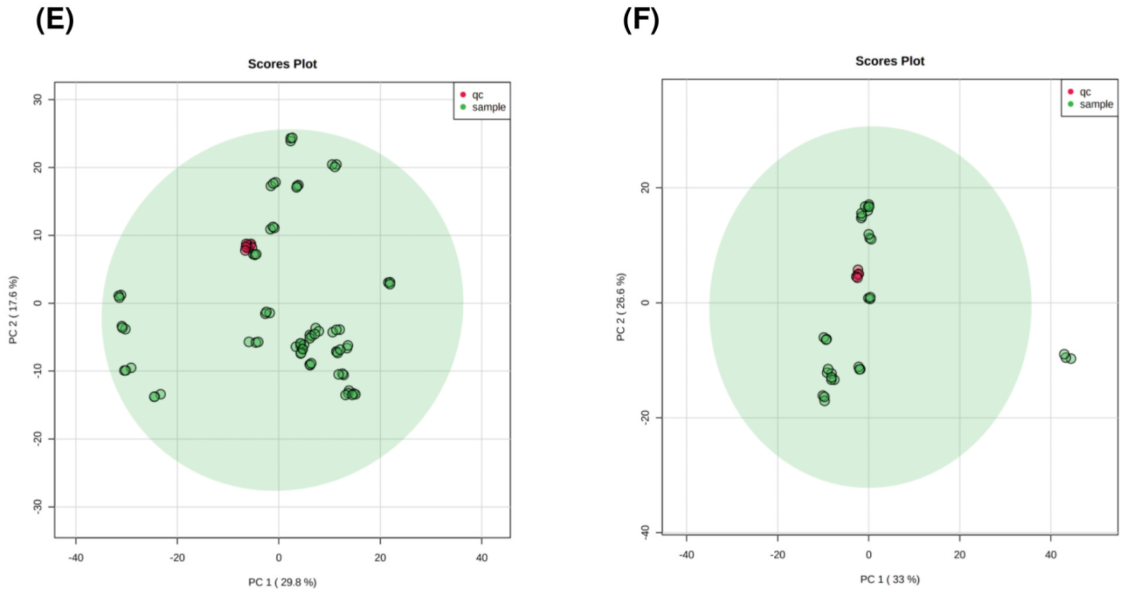

Figure S2. PCA score plot of samples in run 1 – 6 for negative ion mode. Run 1 to 6 (A to F). The QC samples in run 1 were scattered into 2 regions, implying existence of batch effects. QC samples of other runs were tightly clustered together within the respective runs, implying the runs were stable and reliable. Red dot is QC sample; green dot is plasma sample; shade area is the 95% confidence region.

Table S1. Number of total MFs detected, significant MFs, and significant metabolites with level 2 confidence annotation.

**Number**	**Association with gender**[a]		**Association with age**[b]		**Association with age in men**		**Association with age in women**	
Ion mode	+	-	+	-	+	-	+	-
Total MFs detected	2424	834	2424	834	2424	834	2424	834
Significant MFs (FDR < 0.05)	194	90	1020	325	744	225	692	228
Significant metabolites annotated at level 2	15	8	45	16	27	17	25	13
Total significant metabolites annotated*	23		58 (3)		40 (4)		36 (2)	
Total significant metabolites annotated* with QC CV < 30%	18		26		20		16	

MFs: metabolite features; FDR: false discovery rate; [a] adjusted for age; [b] adjusted for gender; () : number of repeated metabolites in both modes; * after subtracting those repeated metabolites that were detected in both modes; QC CV: coefficient of variation for QC.
